# The earliest settlers of Mesoamerica date back to the late Pleistocene

**DOI:** 10.1371/journal.pone.0183345

**Published:** 2017-08-30

**Authors:** Wolfgang Stinnesbeck, Julia Becker, Fabio Hering, Eberhard Frey, Arturo González González, Jens Fohlmeister, Sarah Stinnesbeck, Norbert Frank, Alejandro Terrazas Mata, Martha Elena Benavente, Jerónimo Avilés Olguín, Eugenio Aceves Núñez, Patrick Zell, Michael Deininger

**Affiliations:** 1 Institut für Geowissenschaften, Universität Heidelberg, Im Neuenheimer Feld 234, Heidelberg, Germany; 2 Institut für Meteorologie und Klimaforschung, Karlsruher Institut für Technologie, H.-v.-Helmholtz-Platz 1, Leopoldshafen, Germany; 3 Staatliches Museum für Naturkunde Karlsruhe, Geowissenschaftliche Abteilung, Erbprinzstrasse 13, Karlsuhe; 4 Museo del Desierto, Carlos Abedrop Dávila 3745, Nuevo Centro Metropolitano de Saltillo, Saltillo, Coahuila, Mexico; 5 Institut für Erd- und Umweltwissenschaften, Universität Potsdam, Karl-Liebknecht-Str. 24, Potsdam, Germany; 6 Institut für Umweltphysik, Universität Heidelberg, Im Neuenheimer Feld 229, Heidelberg, Germany; 7 Área de Prehistoria y Evolución del Instituto de Investigaciones Antropológicas de la Universidad Nacional Autónoma de México (UNAM), Mexico; 8 Instituto de la Prehistoria de América, Carretera federal 307, Solidaridad, Solidaridad, Quintana Roo, Mexico; 9 Hessisches Landesmuseum Darmstadt, Friedensplatz 1, Darmstadt, Germany; 10 UCD School of Earth Sciences, University College Dublin, Belfield, Dublin 4, Ireland; Max Planck Institute for the Science of Human History, GERMANY

## Abstract

Preceramic human skeletal remains preserved in submerged caves near Tulum in the Mexican state of Quintana Roo, Mexico, reveal conflicting results regarding ^14^C dating. Here we use U-series techniques for dating a stalagmite overgrowing the pelvis of a human skeleton discovered in the submerged Chan Hol cave. The oldest closed system U/Th age comes from around 21 mm above the pelvis defining the terminus *ante quem* for the pelvis to 11311±370 y BP. However, the skeleton might be considerable older, probably as old as 13 ky BP as indicated by the speleothem stable isotope data. The Chan Hol individual confirms a late Pleistocene settling of Mesoamerica and represents one of the oldest human osteological remains in America.

## Introduction

The early settlement of the Americas is a controversial subject. While genetic evidence suggests a Beringian origin of the earliest inhabitants of the continent [1–5], migration routes used for the southward spread of these humans and the timing of human arrival across the Americas are presently reevaluated [6–11]. The hypothesis of a routing across the exposed Bering land bridge through an ice-free corridor between retreating North American glaciers, at about 12.6 thousand years (ky) ago [12], is increasingly challenged by the discovery of evidence predating the earliest North American widespread archaeological complex, the Clovis culture [13–15]. Based on new sites in both North and South America this emerging consensus suggests that people must have arrived in North America as early as 22 ky ago (e.g. [2, 6, 10, 16–18].

Osteological evidence for early American settlers is scarce and majorly fragmentary, with at present only a few individuals, from North-, Central- and South America, securely predating 11 ky BP [19–24]. Recently, Chatters et al. [25] documented a well-preserved prehistoric skull of a young girl from the submerged Hoyo Negro (Black Hole) sinkhole of the Tulum area, southern Mexico. The individual was ^14^C-dated to 10976±20 y BP (12910–11750 cal y BP) based on bioapatite from tooth enamel [25]. Previously, González González et al. [20, 21] published a similar ^14^C age for a human skeleton from the nearby Naharon cave, also located close to Tulum (11670±60 ^14^C y BP; 13721–13354 y cal BP). These two skeletons belong to the oldest ^14^C-dated New World *Homo sapiens*. They emphasize the enormous potential of the Tulum system of submerged caves as an archive for the human settlement history in America.

**Fig 1:**
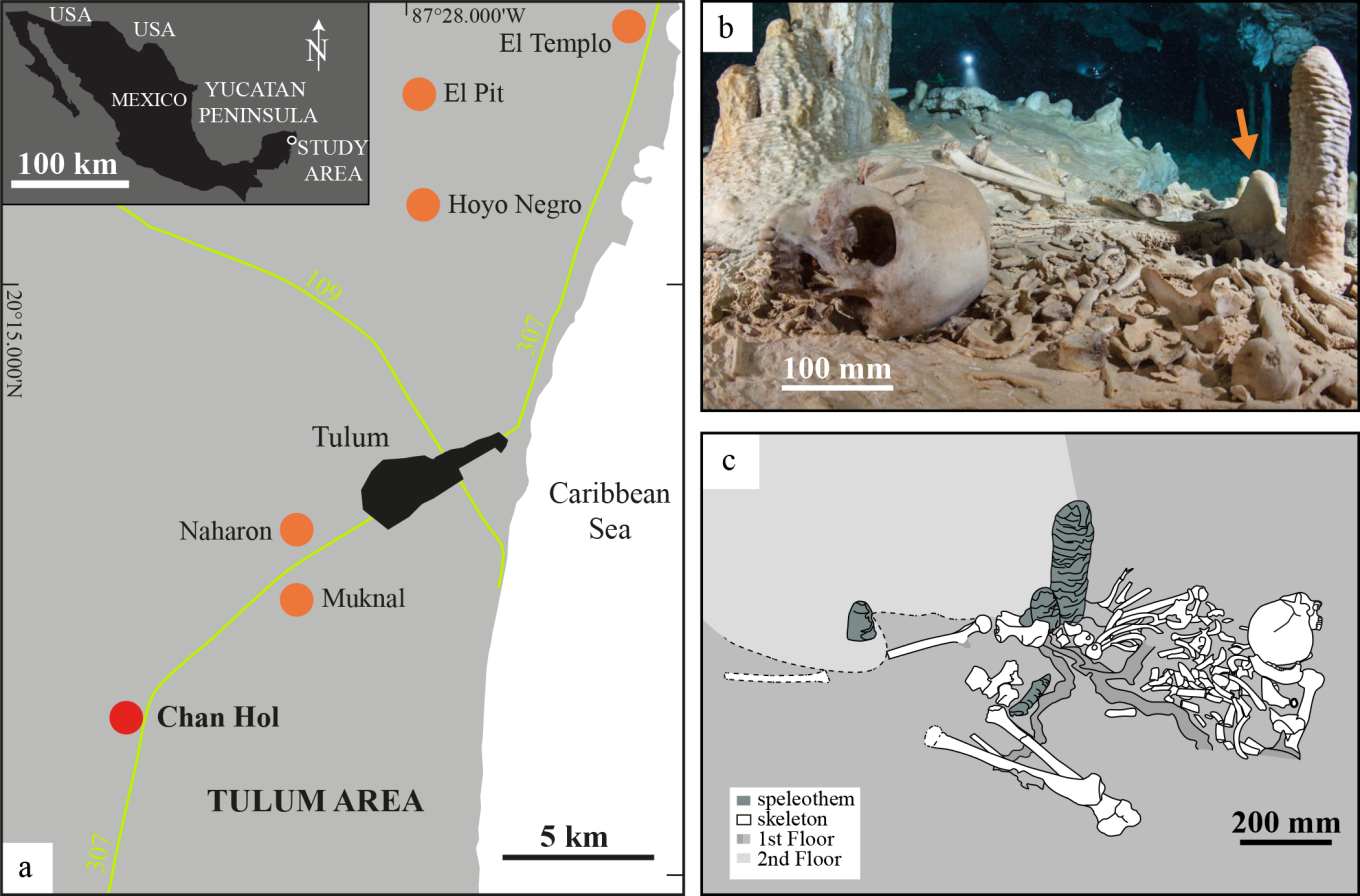
Geographical position and site of the Chan Hol skeleton. *a*: Location of submerged caves containing human skeletal remains dating to >8000 BP in the Tulum area of Quintana Roo, Mexico. Orange dots refer to human remains [20, 21, 25]. The red dot marks Chan Hol II. *b*: The Chan Hol II archaeological site prior to looting. The arrow points to the CH-7 stalagmite analyzed here. (*c*) Reconstruction of the skeleton based on photographs of the site prior to looting. Note that the skeleton was originally complete and almost articulated (Photo courtesy Nick Poole and Thomas Spamberg).

Nevertheless, ^14^C data from the Tulum submerged caves must be considered with extreme caution because the amount of collagen in both bones and teeth of these individuals is extremely low. This lack of collagen is the result of the exposure of the osteological remains for thousands of years to alternating salt- and fresh water environments [21, 25–27]. In addition, bioapatite is highly susceptible to contamination with fossil carbon resulting in false, mostly older ages [25], apart from a general problem with radiocarbon ages in the tropics [26]. Here we use U-series techniques to date a human skeleton that was discovered in the Chan Hol cave near Tulum (here referred to as Chan Hol II, because there are other prehistoric human skeletons in this cave [21]. The pelvis of this skeleton, previously documented briefly by [21], is overgrown by a stalagmite (CH-7; Fig 1).

### Regional setting

The Tulum submerged cave system developed in almost horizontally layered, thick-bedded, shallow-water carbonate bedrock of Neogene ages [28, 29] and is the result of intensive karstification during the Pleistocene [30], caused by a series of sea-level oscillations and changes in the overall hydrology of the area. During Pleistocene stadials, sea-level was more than 100 m below the recent level [31, 32], thus leaving large parts of the cave system dry and accessible. In contrast, during interstadials of the Pleistocene and the early Holocene, between 13 and 7.6 ky BP [33], the karst labyrinth was flooded preserving both archaeological and climate archives. Recent water levels were reached at approximately 4500 y BP [34], although oscillations of up to a few meters are known to have occurred during Maya times [35–37]. The Tulum cave system contains a coastal density stratified aquifer with a freshwater layer overlying penetrating seawater. The depth of the halocline depends on the global sea-level as well as on the thickness of the superimposed freshwater layer and is controlled by the distance to the coastline as well as the amount of precipitation, with a hydraulic gradient across the Yucatan Peninsula (YP) of between 0.5 and 100 mm km^-1^ [38–40]. Water level in the Tulum area is thus approximately equivalent to mean sea-level. Sea-level rise on the YP was predominantly controlled by eustasy, because the peninsula is tectonically stable and glacial isostatic adjustments in this tropical area were minor (e.g. [32, 40, 41]).

### The Chan Hol II site and the skeleton

The Chan Hol II skeleton was located at 20°9.467' N, 87°34.165' W, 15 km southwest of Tulum, Quintana Roo, southern Mexico, and about 11.5 km from the coast line (Fig 1). It was discovered in a low cave tunnel approximately 1240 m southwest of the Chan Hol sinkhole, at about 8.5 m water depth. The maximum depth of the Chan Hol cave is about 13 m below present day sea-level. The halocline is at a depth of about 9 m. Contrary to most other preceramic human skeletons discovered so far in the submerged caves of Tulum, which have been located at water depths of 20 to >30 m [20, 21, 25], the shallow Chan Hol cave must have been accessible up to the middle Holocene [33, 34]. This interpretation is supported by an U/Th age of 5700 y BP of a stalagmite tip from the 8.5 m depth level at Chan Hol. For the nearby Outland cave, [42] postulate a flooding initiating from 8100 cal y BP to a complete inundation at around 6000 cal y BP, which agrees with the results provided here.

Fossil remains were also discovered by us in the extended Chan Hol cave system, though not close to the Chan Hol II skeleton. They include isolated bones of a megalonychid ground sloth, and of extant pacas (*Dasyprocta punctata*), spider monkeys (*Ateles geoffroyi*), peccaries (cf. *Tayassu tajacu*) and white-tailed deer (*Odocoileus virginianus*).

**Fig 2:**
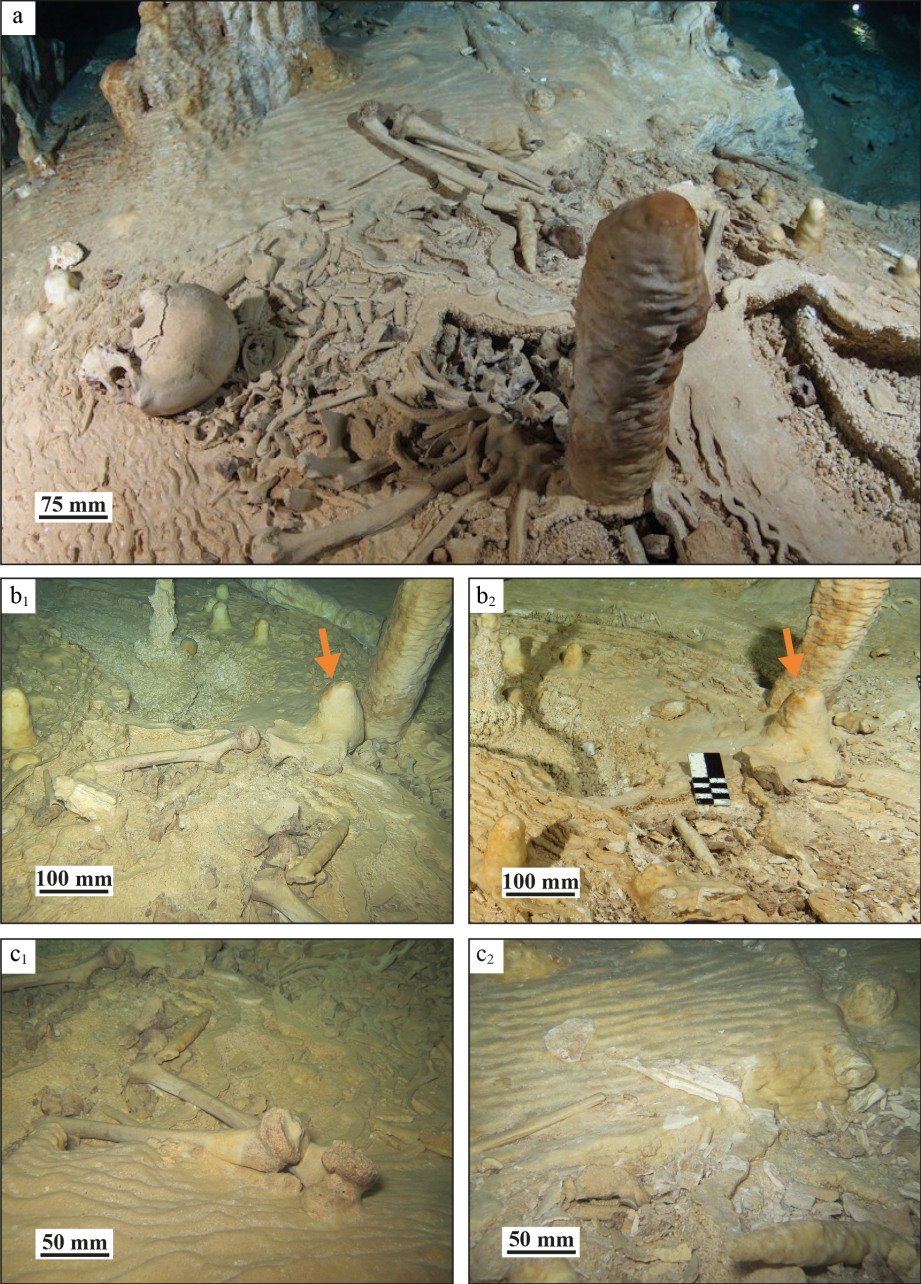
The original Chan Hol II skeleton. Chan Hol II site prior to (*a*, *b*_*1*_.*c*_*1*_) and after (*b*_*2*_, *c*_*2*_) looting. About 10% of the skeleton remained on site, including the pelvis covered by stalagmite CH-7 (red arrows in Figs *b*_*1*_ and *b*_*2*_).

The Chan Hol II skeleton was brought to our knowledge (JAO) in February 2012 through photos in social media. Soon after, the site was vandalized between the 16^th^ and 23^rd^ of March 2012 and all easily collectable bones were stolen. Photographs of the Chan Hol II skeleton prior to this vandalism provide strong evidence that the skeleton must have been more than 80% complete with the skull excellently preserved (Figs 1B, 1C and 2).

The photographs also allow us to reconstruct the original position of the skeleton and indicate that it was preserved nearly articulated (Fig 1B), with the corpse lying on its back. This is concluded from the ribs covering the vertebral column (Figs 1B and 2A) and the position of the left angled femur showing its caudal face (Fig 2B_1_ and 2C_1_). The head was inclined slightly to the right. The right leg was fully extended, while the left leg was flexed at the knee at an angle of 20° (Fig 1C). The right femur was still in an articulated position with the pelvis. Based on these data we speculate that the Chan Hol II human died in the cave and that it was not intentionally buried, but there is no positive evidence for this interpretation. Also, no artifacts were identified close to the skeleton on the photographs of the original site or during our collection.

**Fig 3:**
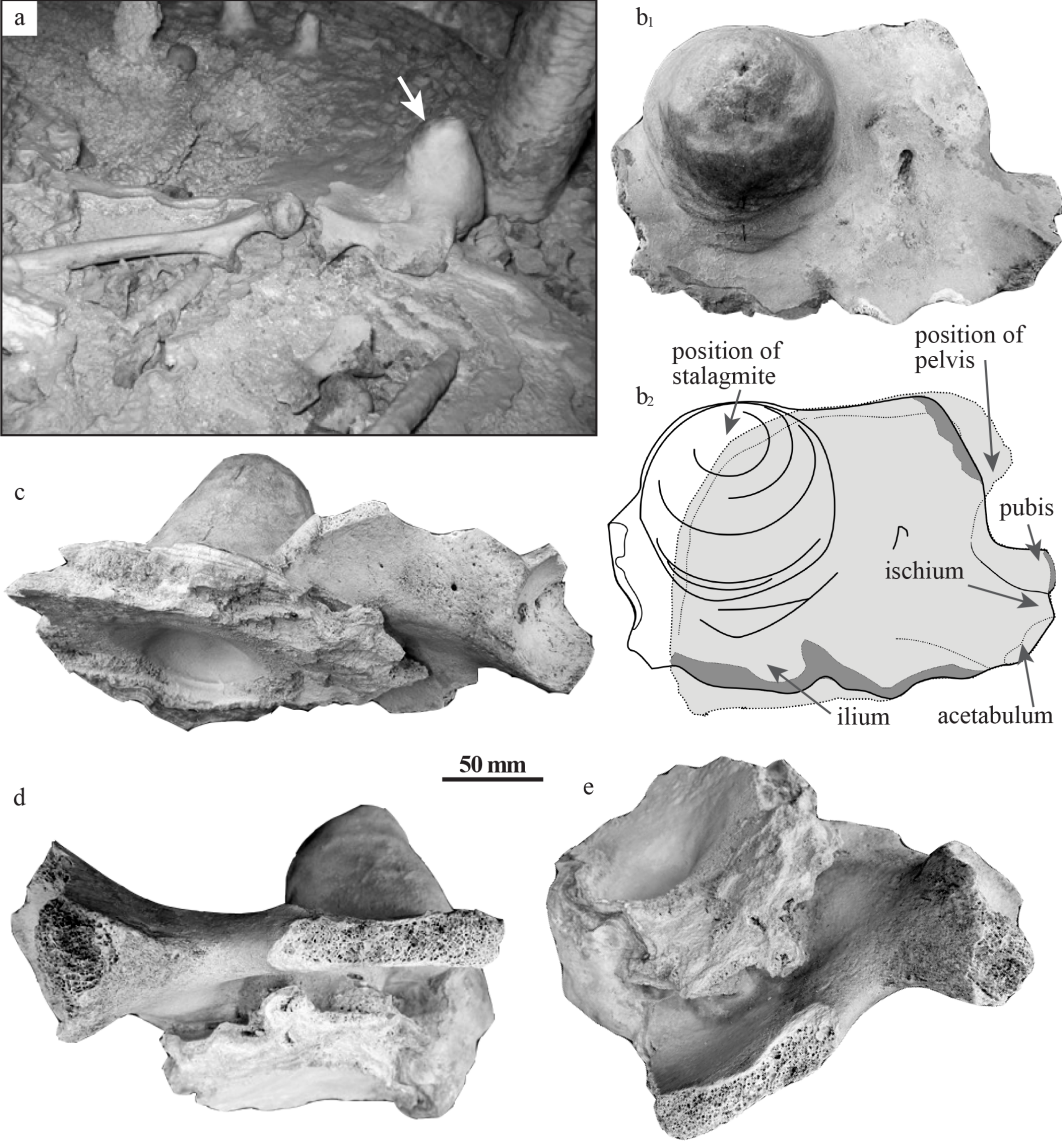
The Chan Hol II pelvis. Different views of the Chan Hol II pelvis within the CH-7 stalagmite. Arrow in Fig 3A points to the CH-7 stalagmite prior to the robbery of the skeleton. Note that the pelvis was then articulated to the right femur. Extraction of collagen and thus ^14^C age determination failed on the bones of the Chan Hol II individual due to the complete dissolution of organic matter, specifically collagen.

After the looting of Chan Hol II only about 10% of the skeleton remained on site (Fig 2). Among the 155 bone fragments collected there are two auditive labyrinths, an incus, fragments of the temporal, the occipital condyle, four teeth (two incisiors, two molars), a mandibular fragment, the hyoid, numerous ribs, carpals and metacarpals, the right pelvis, a patella, tarsals and metatarsals. Embedding of the pelvis in a stalagmite (CH-7) likely prevented this bone from being stolen (Fig 3). Interpretation of sex and age of the Chan Hol II human is speculative, given that our collection only consists of highly fragmentary bones and a few photographs from the original site. We suggest that the individual was a young adult based on the osteophytes in the vertebral bodies, eruption of the third molar in the right half of the mandible, and an epiphysis that was completely fused. Based on the sciatic notch, it was likely a male.

## Results

### The CH-7 stalagmite

**Fig 4:**
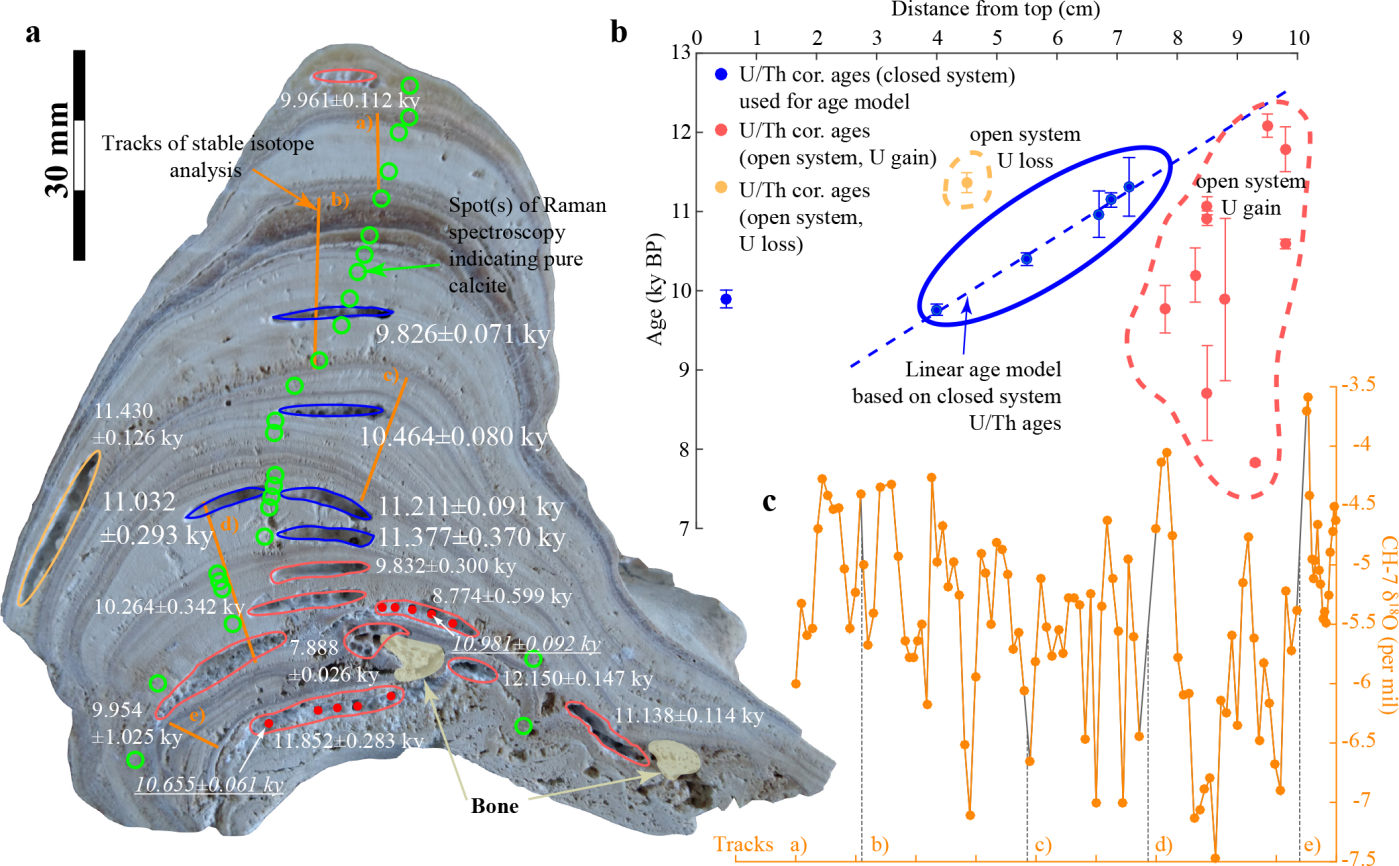
Cross-section of CH-7 and results of U/Th age measurements. Samples for U/Th age measurements are taken along growth layers and are highlighted by coloured frames; red dots within red frames and underlined ages indicate replicates that were drilled into CH-7. Green circles indicate the location where the mineralogy of CH-7 was tested by Raman spectroscopy. Orange lines indicate the location of points, where samples for isotope analysis where taken. (*a*) U/Th ages (y before 2016) above 72 mm from the top (blue) are most likely unaffected by the presence of the encrusted bone and infiltrating seawater, whereas U/Th ages below (red) are likely affected by a re-mineralization processes, infiltrating of the porous encrusted bone, possibly leading to an open-system U series. The orange circle highlights one sample from the steeply sloped and porous edge of the stalagmite, which has been taken to test the possible influence of stalagmite—sea water interactions through time. Please note that ages are given as age before the year of measurement, which is 2016. Ages thus appear older by 66 years than ages given in years BP (i.e. before 1950) as used in Fig 4b and all other figures and text. (*b*) Based on most probable closed-system U/Th ages (see text) it is concluded that the pelvis of the Chan Hol II skeleton dates to a minimum age of 11311±370 y BP. Underlined ages are from samples drilled into the CH-7 stalagmite. All other ages are from samples taken from stalagmite growth layers. (*c*) Stable oxygen isotope profile of CH-7. The labelled section a) to e) corresponds to the isotope tracks shown in (*a*).

Stalagmite CH-7 is 107 mm high with a mean diameter of ~70 mm (Figs 3 and 4). It encloses the human pelvis of the Chan Hol II skeleton at ~95 mm below the top of the stalagmite. The bone is under- and overlain by brown-colored calcareous stalagmite layers, which are each between 1 and 3 mm thick. The internal section of the stalagmite exhibits a succession of milky white with less frequent dark brown calcitic laminae along its long axis. Layers underlying the 3–5 mm thick solid layers below the bone show a wide range of porosity, resembling lime tufa, and they are thus distinct from the dense overlying layers. In addition, the underlying layers are irregularly flexed and bent, which is not seen directly below and above the pelvic fragment, where the lime layers are substantially more homogenous.

### U/Th analysis

17 samples were taken along the growth axis of stalagmite CH-7 and were dated using mass spectrometric measurements of the natural disequilibrium isotope ratios of U and Th [43]. U concentrations of these samples are variable with values ranging between 0.2605 and 12.78 ppm. Initial δ^234^U values are close to the secular equilibrium with values ranging between -33.7‰ and +23.4‰ (Table 1).

**Table 1:** U/Th measurements of the CH-7 stalagmite from Chan Hol cave. Errors are 2σ analytical errors. Corrected ^230^Th ages assume an initial ^230^Th/^232^Th concentration ratio of 3.8 ± 1.9. Please note that corrected ages are given as age (ka) before the year of measurement, which is 2016. Ages thus appear older by 66 years than ages given in years BP (i.e. before 1950) used in all other figures and text.

Lab. Nr.	238U	Error	232Th	Error	230Th/238U	Error	230Th/ 232Th	Error	d^234^U corr.	Error 2σ	Age (uncorr.)	Error	Age (corr.)	Error	d^234^U (initial)	Error 2σ	dft
	(ng/g)	(abso.)	(ng/g)	(abso.)	(Act.rat.)	(abso.)	(Act.rat.)	(abso.)	(^o^/_oo_)	(abso.) ^o^/_oo_	(ka)	(ka)	(ka)	(ka)	(^o^/_oo_)	(abso.) ^o^/_oo_	cm
7413	411,598	0,044	1,5958	0,0031	0,08787	0,00084	69,50	0,57	-2,7	4,4	10,073	0,096	**9,961**	**0,112**	-2,8	4,5	0,5
7412	538,268	0,038	1,6539	0,0032	0,08650	0,00059	86,45	0,49	-3,4	1,5	9,915	0,058	**9,826**	**0,071**	-3,4	1,5	4
7411	494,252	0,027	2,0906	0,0034	0,09294	0,00065	67,48	0,31	6,0	1,4	10,585	0,051	**10,464**	**0,080**	6,2	1,4	5,5
7295	590,553	0,037	4,6726	0,0086	0,10091	0,00102	39,17	0,17	-3,4	1,2	11,659	0,050	**11,430**	**0,126**	-3,6	1,2	4,5
7707	301,23	0,22	1,263	0,025	0,0979	0,0023	71,7	2,2	8,8	5,1	11,151	0,279	**11,032**	**0,293**	9,1	5,3	6,7
7410	353,658	0,021	1,2969	0,0030	0,09989	0,00075	83,65	0,54	14,6	1,5	11,315	0,074	**11,211**	**0,091**	15,1	1,5	6,9
7708	260,51	0,12	1,205	0,022	0,1011	0,0030	69,3	2,3	10,9	4,7	11,509	0,349	**11,377**	**0,370**	11,2	4,9	7,2
7709	331,185	0,089	0,4032	0,0082	0,0877	0,0026	226,0	8,0	15,5	3,2	9,867	0,304	**9,832**	**0,300**	15,9	3,3	7,8
7710	344,57	0,14	0,806	0,016	0,0922	0,0029	121,9	4,4	21,7	4,8	10,330	0,340	**10,264**	**0,342**	22,3	4,9	8,3
6331	273,543	0,470	0,4578	0,0081	0,15666	0,01037	144,84	9,87	19,9	6,1	8,821	0,613	**8,774**	**0,599**	20,4	6,2	8,5
7408	385,346	0,026	1,2234	0,0022	0,09791	0,00073	94,96	0,62	15,3	2,1	11,071	0,078	**10,981**	**0,092**	15,8	2,2	8,5
6282	288,507	0,433	0,3907	0,0043	0,08857	0,00854	200,61	19,39	16,3	14,3	9,992	0,996	**9,954**	**1,025**	16,8	15,0	8,8
7296	1386,210	0,070	10,3090	0,0159	0,09703	0,00093	40,07	0,13	-17,4	0,6	11,356	0,034	**11,138**	**0,114**	-18,0	0,6	8,5
7409	12779,036	0,703	9,4110	0,0209	0,06755	0,00021	282,14	1,01	-32,9	0,4	7,910	0,023	**7,888**	**0,026**	-33,7	0,4	9,3
6321	706,188	1,202	0,6430	0,0062	0,10457	0,00233	349,33	8,43	16,6	3,9	11,877	0,280	**11,852**	**0,283**	17,2	4,0	9,8
7407	1800,994	0,102	5,6876	0,0109	0,09248	0,00051	90,00	0,37	-13,1	0,5	10,747	0,043	**10,655**	**0,061**	-13,5	0,6	9,8
6396	712,213	1,135	2,2923	0,0107	0,10764	0,00112	103,66	1,16	22,7	3,9	12,240	0,139	**12,150**	**0,147**	23,4	4,0	9,5

Contamination of the samples with non-carbonate particles is mostly insignificant. This is indicated by ^232^Th concentrations of <10 ng g-1, introducing only minor age corrections when using the bulk Earth ^230^Th/^232^Th ratio as a correlate [43]. Thus, corrected U/Th ages for CH-7 are variable, ranging between 7.82 ky BP and 12.09 ky BP with most data, however, clustering between 9.8 and 12.1 ky (Fig 4A, Table 1).

**Fig 5:**
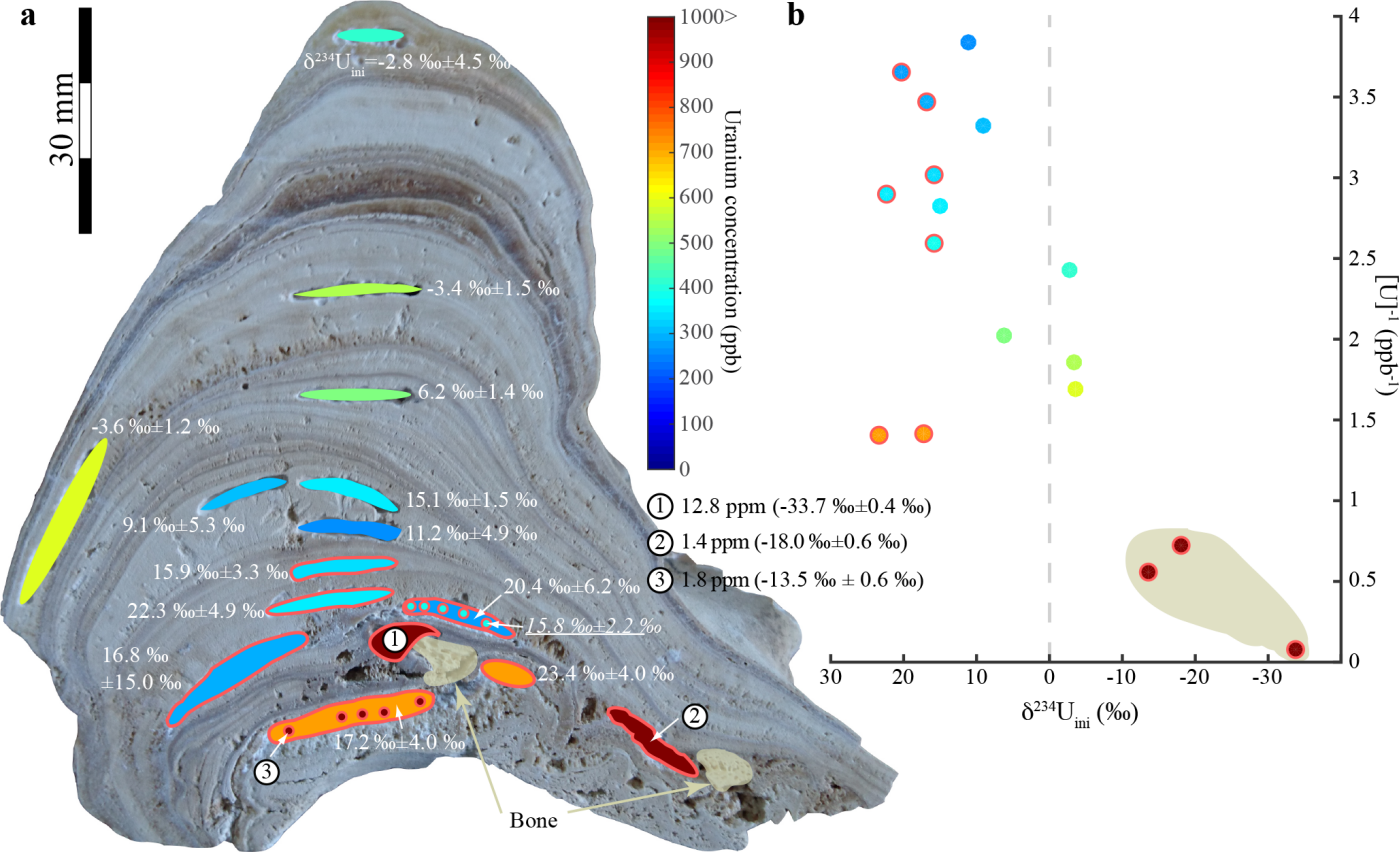
Uranium concentrations and initial δ^234^U values of CH-7. The U concentration of samples analysed by us is illustrated by warm/cool coloured frames indicating higher/lower U concentrations (a) Samples 1–3 present the highest U concentrations and lowest initial δ^234^U values. (b) The relationship between the U concentrations and the initial δ^234^U values show that samples with highest U concentrations are generally low in initial δ^234^U. This is likely caused by the influence of the pelvic bone, causing a U gain and at the same time a decrease in ^234^U/^238^U.

Only the calculated U-Th ages of samples taken from the stalagmite growth axis above 72 mm can be interpreted as in stratigraphic order and reflecting a closed system behavior. The U-Th ages of samples below the 72 mm cluster reveal much younger ages than those above. This contradicts a quasi linear growth model for the stalagmite. Furthermore, the U-Th age of a sample at the left flank of CH-7 at 45 mm distance from top (dft) is much older compared to this linear age-depth relationship (Fig 4B). The relationship between the U concentration and the initial δ^234^U isotopy is asymptotic between these two parameters, indicating that δ^234^U decreases for higher U concentrations and proximity to the pelvis. The highest U concentrations, which reach 12.8 ppm, and smallest δ^234^U values are measured for samples that are adjacent to the pelvis (Fig 5).

It is a matter of fact that the U concentration increases in bones post mortem to as high as 100 ppm (e.g. [44]). This is due to the “soaking up effect” of U into the bone due to diffusion (e.g. [44]). One approach to date a bone or tooth by U-series is to use this effect and to determine the U-isotopes of subsamples across the bone by application of an adsorption-diffusion (D-A) model (e.g. [45, 46]). Because of the porous structure of the pelvis, which makes it likely that pore water in the spongiosa disturbed the original D-A relationship, this approach was not adopted here. Instead, we identified the *termini ante quem* and *post quem*, by using the reliable closed system U/Th ages of carbonate above and below the pelvis from the overgrowing stalagmite, respectively. While the stalagmite base around the pelvic bone fragment is characterized by a complex morphological texture, as well as age which complicate the determination of the terminus *post quem* and the use of these ages, determination of the terminus *ante quem* is possible from ages above the pelvis. These show a linear age-depth model and likely no influence on the U- and Th-system (Fig 5).

The exceedingly porous carbonate texture adjacent to and below the pelvis is likely influenced by U diffusion, as indicated by extremely high U concentrations. These high U concentrations result from an opening of the uptake series system, for which the source of U is unlikely seawater because the δ^234^U values stay in close ranges. Hence, it could be that U is redistributed from the very local environment resulting in variable ages, such as the very young age of the sample in direct contact with the bone. Most likely, this effect is restricted to the lowermost section of the stalagmite below 72 mm (dft). We cannot exclude the possibility that samples above 72 mm (dft) are also influenced by U-exchange, but the low variability and quasi linear age-depth relationship suggests that this effect is likely negligible and within ranges of standard age-uncertainty. Based on U/Th ages above 72 mm of the CH-7 stalagmite the minimum *terminus ante quem* of the pelvis is 11311±370 y BP.

Regarding the significantly older age of the sample at the flank of CH-7, one must assume U loss possibly due to the mineralogical change from presumably aragonite to calcite, as is indicated by the needle like texture and present day calcite configuration.

### Stable isotope analysis

**Fig 6:**
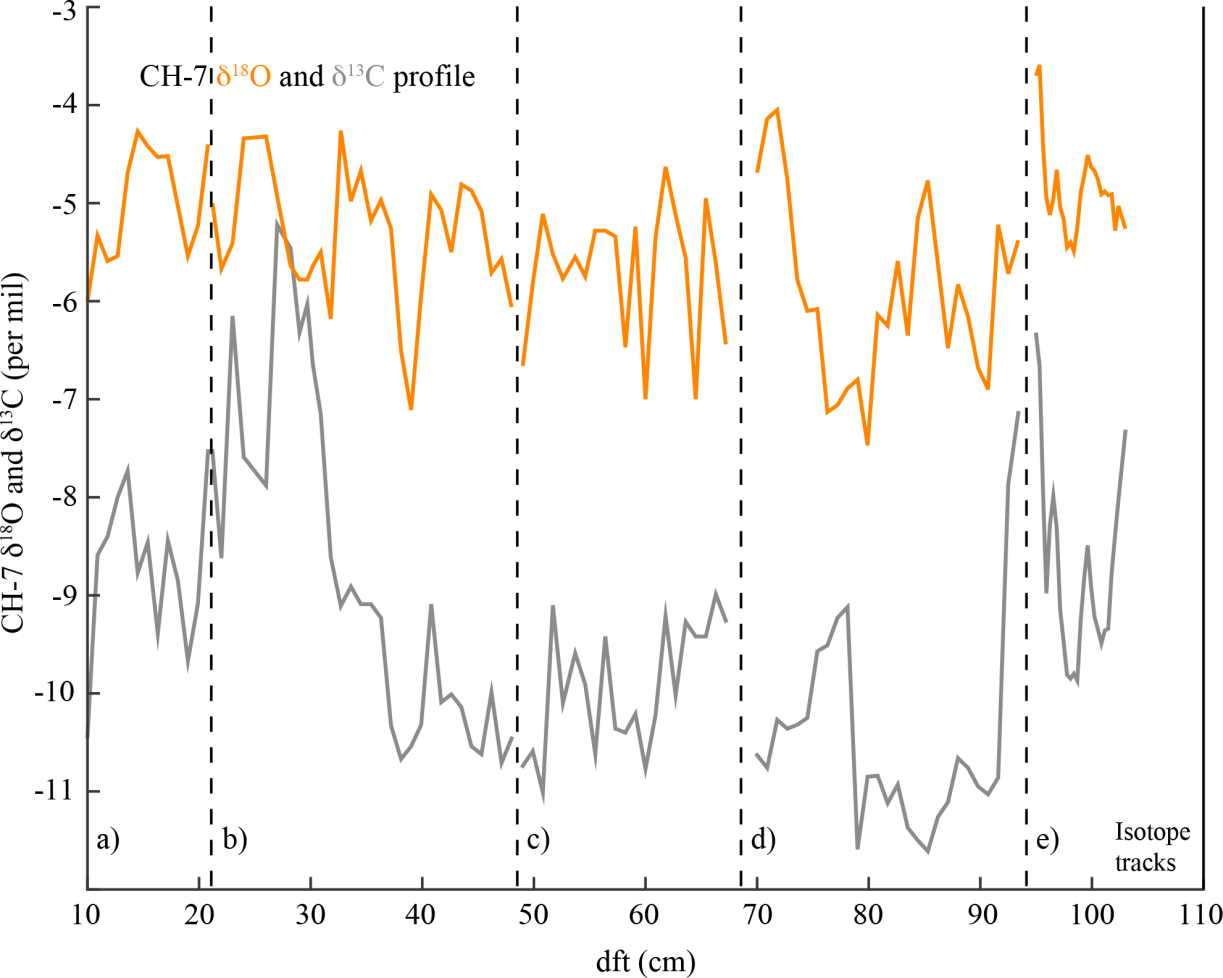
Stable isotope profile. Stable oxygen and carbon isotope profile of CH-7 (δ^18^O and δ^13^C, orange and grey curve, respectively).

Stable oxygen and carbon isotope values, δ^18^O and δ^13^C (expressed in the δ-notation relative to V-PDB), vary from -3.59‰ to -7.47‰ and from -5.22‰ to -11.61‰, respectively (Fig 6; S1 Table).

A significant change in the δ^18^O profile of CH-7 occurs in the lower part of CH-7 between 100 mm and 70 mm dft of CH-7. The δ^18^O values vary by as much as 3.87‰ and show a w-shape in this section. Above 70 mm dft, the δ^18^O profile of CH-7 is more variable but a trend towards higher values is identified towards the top of CH-7 being -5.41‰ on average, with a 1-sigma standard deviation of 0.69‰. Internal δ^13^C variability is less compared to that of the δ^18^O. Heaviest δ^13^C values occur at the bottom and top-most isotope tracks, with lighter δ^13^C values in between.

### Age assessment

Our Chan Hol (CH-7) isotope record indicates a very pronounced 4‰ shift across tracks c and d (Fig 6), from the most positive (-3.5‰) δ^18^O values at 95 mm to the most negative (-7.5‰) δ^18^O values at 80 mm and back to -4‰ at around 11.3 ky BP, the oldest open system U-Th date. This near 4‰ excursion presents a significant shift in the isotopic signal.

Our oldest open system U/Th date of 11311±370 y BP coincides with the end of the Younger Dryas (YD), a time episode characterized by a brief return to near glacial conditions interrupting the general amelioration of climate conditions at the last deglaciation [47–50]. The ~ 4‰ shift in our CH-7 δ^18^O record occurs across a 21 mm interval below this last U/Th date, and hence potentially falls into the time interval of the YD. Seen the amplitude and signature of the CH-7 δ^18^O signal, it seems likely that the YD, or part of the YD time interval, is displayed in our Chan Hol speleothem. As we do not have an absolute (U/Th) date in this lower part of the speleothem we can only, as a first guess, assume a continuous growth rate for the CH-7 stalagmite in the interval below our last independent U/Th date and linearly extrapolate our age model back in time. We can then compare our stable isotope record of CH-7 to other well-known independently dated climate archives from a similar time interval and area.

**Fig 7:**
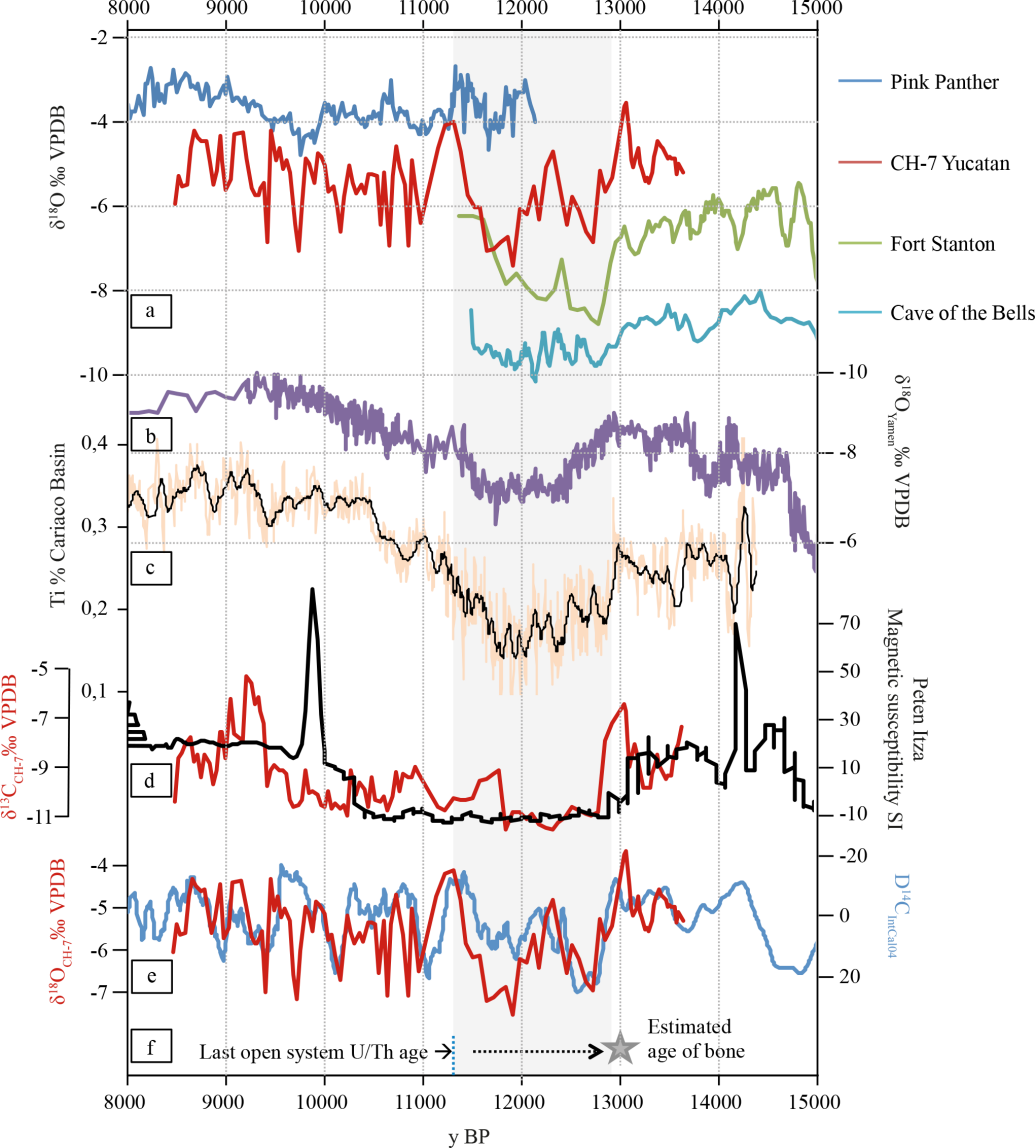
Comparison of oxygen isotope and other environmental data across the Pleistocene-Holocene boundary. a) Speleothem δ^18^O data (blue, red, green and light blue curve, respectively) of Pink Panther cave, Guadalupe Mountains, New Mexico, USA [51], Chan Hol cave (this study), Fort Stanton cave, New Mexico, USA [52] and Cave of the Bells, Arizona, USA [53]. b) China deglacial speleothem oxygen isotope data from Yamen Cave, Guizhou Province, China, [56] on a reversed scale; c) Cariaco Basin, Venezuela, Ti % (orange curve) overlain by moving average (black curve) [57]; d) comparison of Peten Itza magnetic susceptibility (black curve) [58] and CH-7 δ^13^C data (red curve); e) Covariation of CH-7 δ^18^O record (red line) and residual ∆^14^C (blue line) 1000 year moving average of IntCal04 [58–60]. Scales for two records are in opposite direction to each other to show negative correlation. Note that age models of both records are completely independent from each other; f) last closed system U/Th date (blue vertical stippled line). Extrapolation (black stippled arrow) of age model by assuming constant speleothem growth rate and interpretation of CH-7 climate signal estimates the age of the bone to around 12.8 ky (grey asterisk). All data are presented in years BP. Grey shaded interval indicates the Younger Dryas time episode.

Unfortunately, speleothem records covering the YD interval are rare from the wider Caribbean region. The closest records come from New Mexico and Arizona (Fig 7A) and have been discussed in detail by [51–53]. These precipitation sensitive speleothem records have been U/Th dated and interpreted to reflect changes in the intensity of the North American monsoon region during the YD time interval, in concert with global climate as recorded in Greenland Ice cores [54, 55], Asian speleothems (Fig 7B; [56]), or Cariaco Basin (off Venezuela) Ti % (Fig 7C; [57]). In terms of signature, absolute values and amplitude our Yucatan δ^18^O record resembles the American speleothem records and hence reflects a similar climate signal. It further resembles the global climate signal during the YD time interval as reflected in the Yamen speleothem (China) δ^18^O and Caricao Basin Ti % records (Fig 7B and 7C). Therefore, we are confident that the Chan Hol stalagmite has indeed grown throughout the YD time interval.

Confirmation for this age assessment comes from the comparison of the CH-7 carbon isotope record with radio carbon dated Lake Peten Itza (Guatemala) magnetic susceptibility [58] (Fig 7D). Variations in the Lake Peten Itza magnetic susceptibility record reflect changes in the sediment lithology, with high values associated with clay-rich horizons and low values associated with gypsum deposits, thereby representing episodes of high and low lake levels, respectively [61].

Speleothem carbon isotope values depend on the amount of local rainfall and infiltration of vegetation cover. During the late Pleistocene, the Tulum area was dominated by ‘steppe’ and possibly looked like the Irish Burren today [62]. The dense tropical forest that dominates the present-day landscape only developed around 9000 years ago [63–65]. Due to the low sea-level, local rainfall at the time of CH-7 growth would have immediately infiltrated the epikarst, with water level in the karst caves dependent on the amount of rainfall. Consequently, the CH-7 δ^13^C record should reflect the availability of CO_2_ (δ^13^C≈-10 per mil) during the dissolution of limestone in the epikarst (δ^13^C≈0 per mil), with decreasing (increasing) δ^13^C of CH-7 reflecting lower (higher) infiltration, respectively. In this way, both the Peten Itza and CH-7 records reflect changes in water runoff into the lake and cave, respectively, with lowest run off signals during the early YD. This is also similar to the Cariaco Basin Ti % record, where low Ti percentages reflect low river runoff into the basin at that time (Fig 7C; [57]).

Although the proxy signals of both Peten Itza and CH-7 contain a certain portion of nonlinear response to vegetation cover retaining water and CO_2_ dissolution levels in the epikarst and lake water, respectively, the agreement between the two different records is good. Both records show coeval episodes of low lake stand (Peten Itza) and infiltration (CH-7), and high lakes stand and infiltration, respectively, across the growth interval of CH-7.

A last confirmation for our age assessment comes from comparison of the CH-7 oxygen isotope record with the detrended residual Δ^14^C data [59–60]. This comparison, presented in Fig 7E, is inspired by [51] who showed a correlation between the δ^18^O variability of Pink Panther cave and the detrended residual Δ^14^C data, postulating a linkage of North American monsoonal precipitation and solar forcing through modulation in the Walker circulation, and the tropical Pacific Decadal Oscillation and El Niño–Southern Oscillation systems [51]. From the visual inspection, CH-7 δ^18^O and detrended residual Δ^14^C data show a remarkable similarity, with episodes of increased solar activity expressed as negative Δ^14^C, well correlated with positive δ^18^O anomalies in CH-7. Correlation is reasonably high (0.48 for inversed δ^18^O) considering the different nature of the proxies and the independence of the age models.

To summarize, we are confident that our age assessment is reasonably correct and that the YD time interval is indeed recorded in our CH-7 speleothem. This rises the age of the pelvis from the U/Th derived *terminus ante quem* of 11311 y BP to an age as old as 13 kyr BP (Fig 7F).

## Discussion

The Chan Hol II individual was discovered at about 1240 meters away from the nearest modern entrance, the Chan Hol sinkhole. The skeleton disarticulated slightly during the final stages of decomposition and, probably again, during the early to middle Holocene flooding of the cave, but most bones still lie close to their original anatomical position. Even small bones, like auditory ossicles, hyoid, or ungual phalanges are present. The person must thus have died in the cave at a time when the cave floor was dry [21]. The decay of the carcass occurred *in-situ*. Growth of the CH-7 stalagmite began after the decay of the Chan Hol II individual was complete. This interpretation is consistent with the macroscopic sequence of basal-most stalagmite laminae. At that time, the drip point was located near the margin of the pelvis (Fig 5). The calcite layer precipitated by the lateral run-off dripping water embraced the pelvic bone from laterally to ventrally, with its ventral surface coalescent with the stalagmite, due to flow extension below the pelvis. This close overgrowth could not have taken place with bones covered by soft tissue. The porous tufa-like layer conforming the basis of the stalagmite must therefore have formed at that time, when the cave was dry and the pelvis completely exposed on the cave floor for an unknown amount of time.

No data are at hand to define the amount of time that elapsed between the death of the individual and initial growth of the CH-7 stalagmite, nor the lapse needed for maceration and decay of this individual under the environmental conditions prevailing in the cave during the YD. Corpses decaying in caves are mostly decomposed by fungi and insects. Both are not able to move bones [66]. According to [66], a 25 kg kangaroo carcass, deposited in a cave in southern Australia, is completely decayed after a little more than 1000 days. For the complete decay of a human carcass with a body mass of 60 kg one would expect a minimum decay time of 2000 days as an estimate. Our U/Th datum of 11311±370 y BP at 72 mm of the CH-7 stalagmite and even the 13 ky BP age assignment resulting from the speleothem stable isotope record must therefore be regarded as minimum ages of the Chan Hol II skeleton.

Validation of our age assessment has been done by comparison of our CH-7 stable isotope data with different independently dated climate records (Fig 7). We stress, that no age correlation or wiggle matching has been carried out to any of the records. All we did was to apply the linear age model from the closed system U/Th dates to the lower part of the stalagmite.

The comparison of our CH-7 stable isotope record with other climate records indicates that the Chan Hol speleothem indeed covers the Younger Dryas time interval (Fig 7). In terms of amplitude and absolute value, the Yucatan δ^18^O record fits well the δ^18^O signal of speleothem records of New Mexico [51–52] and Arizona [53] that have been demonstrated to record the global climate signal of the YD. The American speleothem δ^18^O records have further been interpreted to reflect changes in the contribution, intensity and source of winter versus summer precipitation, the latter being fed from the Caribbean, and these changes have been linked to changes in the positioning of the polar jet stream related to the still northerly expansion of ice sheets causing modulation of winter storm tracks across the continent [52–53]. On the other hand, the relationship between the Pink Panther cave oxygen isotope record and solar forcing has been explained through changes in the Walker circulation and the Pacific Decadal Oscillation and El Niño–Southern Oscillation systems [51] but shows a significant similarity to Northern Hemisphere records. Finally, the climate signals at Peten Itza and Cariaco Basin have been discussed to reflect swings in the position of the ITCZ [57, 58].

In the end, these climate components are all linked to a complex system [67] and likely influenced our Chan Hol record. However, it is beyond the scope of this paper to disentangle the different components of this complex climate system. This deserves a thorough discussion in a separate paper. The focus of the current paper is on the dating of the Chan Hol II skeleton and we can confidentially state that with the U/Th dates and the stable isotope record at hand we can approximate the age of the Chan Hol II individual to ~13 ky BP.

## Conclusions

Speleothem (U/Th) age data indicate that the Chan Hol underwater cave south of Tulum, state of Quintana Roo, Mexico, was accessed by humans during the Younger Dryas period, i.e. during the late Pleistocene. This is indicated by a minimum speleothem age of 11311±370 y BP of a stalagmite encrusting and overgrowing the pelvic bone of an almost articulated human individual in this cave. The age was measured at 72 mm depth from the top of the CH-7 stalagmite, at about 21 mm above the pelvis and 33 mm above the base of the stalagmite, while ages in the immediate bone vicinity are altered due to uranium dissolution. 11311±370 y BP is thus a minimum age for the skeleton. Based on a linear growth model and extension of growth rates from the well-dated upper part of the CH-7 stalagmite to its lower portion and base, the age of the Chan Hol II human rises to ~13 ky BP. The Chan Hol II skeleton thus represents one of the oldest directly dated osteological heritage of a human from the American continent. Age of the Chan Hol II human equals that of other skeletons in the Tulum cave system (e.g. Naia, Najaron), thus emphasizing the importance of these caves for early human settlement in the Americas [20, 21, 25].

## Methods

**Fig 8:**
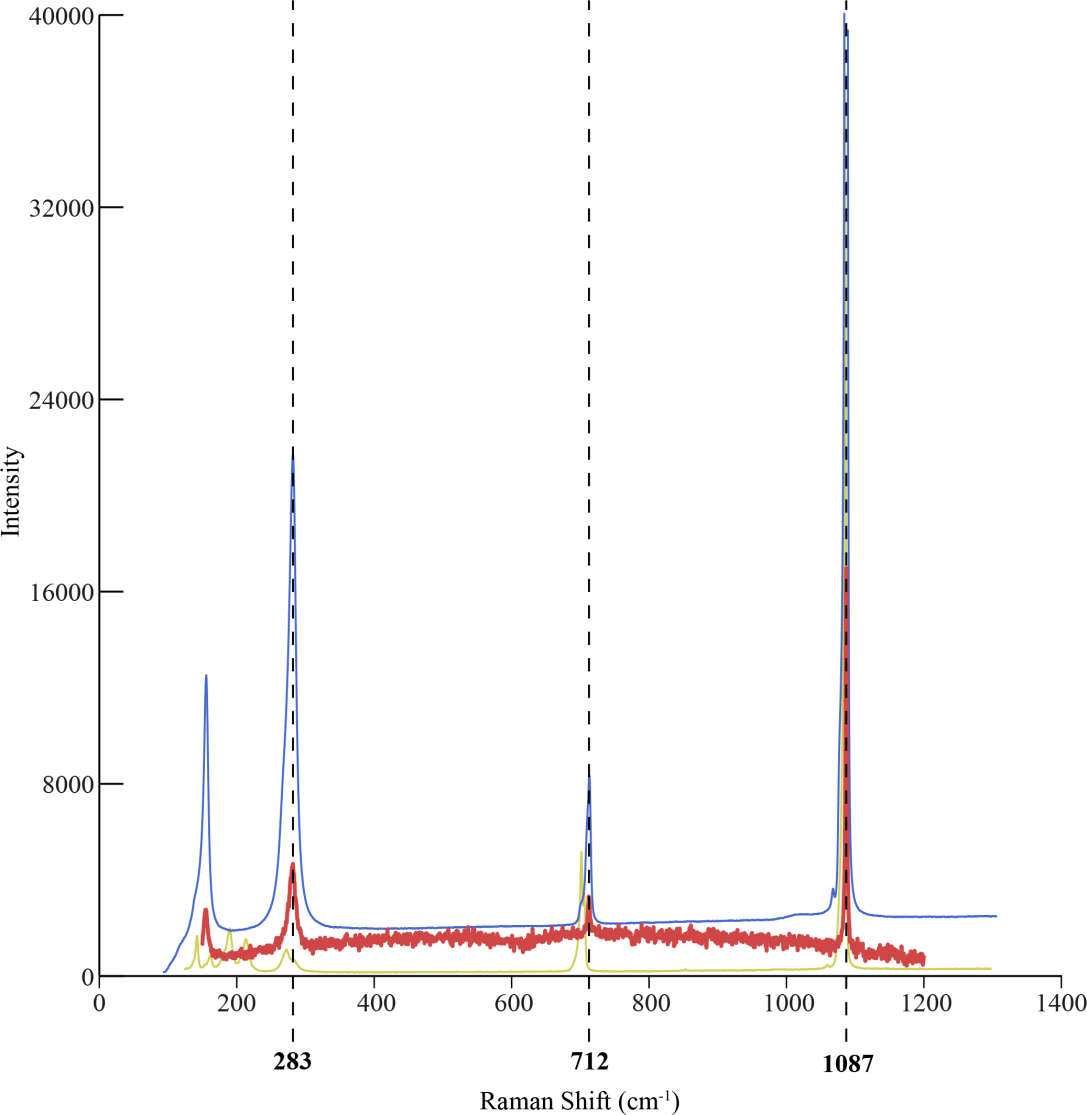
Raman spectrum of CH-7. Values measured are compared to Raman spectra of reference calcite and aragonite (RRUFF database; [68]. Comparison shows that CH-7 consists of calcite.

The CH-7 stalagmite consists of only calcite (no aragonite was detected), as was confirmed by 25 measurements using Raman spectroscopy techniques at the Institute of Earth Sciences at Mainz University, Germany (Figs 5 and 8). For Raman spectroscopy a Horiba Jobin Yvon was used that was connected to an Olympus BX41 microscope using a Nd-YAK laser at a wavelength of 532.12 nm (hole = 400 μm; slit = 100 μm).

### U/Th dating

Samples for U/Th-dating were drilled from stalagmite CH-7 using a Dremel Fortifex precision tool, 1 mm in diameter. The samples were taken along laminar growth layers to minimize mixing of material of different age and thus age uncertainties. Individual sample thickness is typically 2 mm (in growth direction), with an individual sample weight of between 100 and 150 μg. All samples (carbonate powder) were prepared for measurements in the clean laboratories at the Institute of Earth Sciences and Institute of Environmental Physics, both Heidelberg University, by wet-column chemistry using UETVA® resin. All samples were spiked using a Th-U multi-spike. U- and Th-isotopes were analyzed using ICP-MS (Thermo Finnigan Neptune Plus and iCAP (RQ), respectively) at the Institute of Environmental Physics at Heidelberg University. Details for sample preparation and U- and Th-isotope analysis are documented in [69]. Ages were calculated using the half-lives of both elements as determined by [70]. Detrital correction was performed using a bulk Earth value of 3.8 ± 1.9. Age uncertainties are quoted at the 2-σ level and do not include half-life uncertainties. The reference year for all ages given in the study is 1950 AD (i.e. 0 BP).

### U/Th ages and the growth of the CH-7 stalagmite

U/Th ages in the upper 72 mm of the CH-7 stalagmite are approximately consistent with the macroscopic sequence of individual laminae growing onto each other (Fig 4). The basal-most layer was dated to 11363±304 y BP. In this layer, the drip point (the highest point of each stalagmite layer) is located at about 20 mm lateral to the pelvis (Fig 4). The layer embraces the bone from lateral to ventral and its ventral surface is coalescent with the stalagmite. The dripping water accumulated lateral of the bone and ran off diffusing laterally, with the flow expanding below the pelvis. To do so, the body must then have been completely decayed already. In a second step, calcium carbonate-rich water, dripping from the ceiling, accumulated next to the pelvis and enclosed the bone completely. The porous tufa-like layer conforming the base of the stalagmite and dated to >11363 y BP, must therefore have formed when the cave was dry and the carcass already completely skeletonized. This is concluded from the spongy carbonate crust that formed beneath the pelvis, at a time when this bone blank of soft tissue.

### C and O isotope analysis of CH-7 stalagmite

Stable oxygen and carbon isotope samples were micro-milled and measured at the Institute for Earth Sciences (GeoZentrum Nordbayern), Friedrich-Albert-Universität Erlangen, Germany. A total of 117 data points was sampled along five transects, each along the growth axis of stalagmite CH-7 (Figs 4 and 6). A minimum of 0.05 to 0.1 mg CaCO_3_ was analyzed to ensure precise measurement. Carbonate powders were reacted with 100% phosphoric acid at 70°C, using a Gasbench II connected to a ThermoFisher Delta V Plus mass spectrometer All values are reported in per mil relative to V-PDB through international standard NBS19. Reproducibility was monitored by international and in house laboratory standards and was 0.5‰ and 0.8‰ for δ^13^C and δ^18^O, respectively.

## Supporting information

S1 Table Stable isotope (δ^13^O, δ^18^O) measurements of the CH-7 stalagmite from Chan Hol cave. (DOCX)
