# Supplementary material for: The earliest settlers of Mesoamerica date back to the late Pleistocene
Source: PLoS One. 2017 Aug 30;12(8):e0183345. doi: 10.1371/journal.pone.0183345 (PMC5576649; doi:10.1371/journal.pone.0183345)
Supplement: S1 Table — (DOCX) [file pone.0183345.s001.docx]

Isotope d18O d13C Date Identifier 1 Identifier 2

Track depth distance Mean Mean

from top (mm) (mm) V-PDB V-PDB

1 10,00 -6,00 -10,44 08/26/15 CH07-05-2 Heidelberg

1 10,90 0,90 -5,33 -8,59 08/26/15 CH07-05-1.11 Heidelberg

1 11,80 0,90 -5,59 -8,40 08/26/15 CH07-05-1.10 Heidelberg

1 12,70 0,90 -5,54 -8,00 08/26/15 CH07-05-1.9 Heidelberg

1 13,60 0,90 -4,69 -7,73 08/26/15 CH07-05-1.8 Heidelberg

1 14,50 0,90 -4,27 -8,77 08/26/15 CH07-05-1.7 Heidelberg

1 15,40 0,90 -4,42 -8,46 08/26/15 CH07-05-1.6 Heidelberg

1 16,30 0,90 -4,53 -9,39 08/26/15 CH07-05-1.5 Heidelberg

1 17,20 0,90 -4,52 -8,44 08/26/15 CH07-05-1.4 Heidelberg

1 18,10 0,90 -5,03 -8,85 08/26/15 CH07-05-1.3 Heidelberg

1 19,00 0,90 -5,54 -9,67 08/26/15 CH07-05-1.2 Heidelberg

1 19,90 0,90 -5,23 -9,08 08/26/15 CH07-05-1.1 Heidelberg

1 20,80 0,90 -4,40 -7,53 08/26/15 CH07-05-1 Heidelberg

2 21,20 21,20 -5,00 -7,53 08/26/15 CH07-04-31 Heidelberg

2 22,00 0,80 -5,67 -8,62 08/26/15 CH07-04-30 Heidelberg

2 23,00 1,00 -5,41 -6,15 08/26/15 CH07-04-29 Heidelberg

2 24,00 1,00 -4,34 -7,59 08/26/15 CH07-04-28 Heidelberg

2 26,00 2,00 -4,32 -7,88 08/26/15 CH07-04-27 Heidelberg

2 27,00 1,00 -4,93 -5,22 08/26/15 CH07-04-26 Heidelberg

2 28,20 1,20 -5,64 -5,46 08/26/15 CH07-04-25 Heidelberg

2 29,00 0,80 -5,78 -6,32 08/26/15 CH07-04-24 Heidelberg

2 29,70 0,70 -5,78 -6,03 08/26/15 CH07-04-23 Heidelberg

2 30,20 0,50 -5,64 -6,65 08/26/15 CH07-04-22 Heidelberg

2 30,90 0,70 -5,50 -7,15 08/26/15 CH07-04-21 Heidelberg

2 31,80 0,90 -5,45 -7,44 08/26/15 CH07-04-2 Heidelberg

2 31,80 0,00 -6,92 -9,77 08/26/15 CH07-04-1.18 Heidelberg

2 32,70 0,90 -4,26 -9,11 08/26/15 CH07-04-1.17 Heidelberg

2 33,60 0,90 -4,98 -8,91 08/26/15 CH07-04-1.16 Heidelberg

2 34,50 0,90 -4,67 -9,09 08/26/15 CH07-04-1.15 Heidelberg

2 35,40 0,90 -5,18 -9,09 08/26/15 CH07-04-1.14 Heidelberg

2 36,30 0,90 -4,97 -9,23 08/26/15 CH07-04-1.13 Heidelberg

2 37,20 0,90 -5,26 -10,33 08/26/15 CH07-04-1.12 Heidelberg

2 38,10 0,90 -6,51 -10,67 08/26/15 CH07-04-1.11 Heidelberg

2 39,00 0,90 -7,11 -10,54 08/26/15 CH07-04-1.10 Heidelberg

2 39,90 0,90 -5,94 -10,32 08/26/15 CH07-04-1.9 Heidelberg

2 40,80 0,90 -4,91 -9,09 08/26/15 CH07-04-1.8 Heidelberg

2 41,70 0,90 -5,07 -10,09 08/26/15 CH07-04-1.7 Heidelberg

2 42,60 0,90 -5,50 -10,01 08/26/15 CH07-04-1.6 Heidelberg

2 43,50 0,90 -4,81 -10,14 08/26/15 CH07-04-1.5 Heidelberg

2 44,40 0,90 -4,87 -10,54 08/26/15 CH07-04-1.4 Heidelberg

2 45,30 0,90 -5,08 -10,62 08/26/15 CH07-04-1.3 Heidelberg

2 46,20 0,90 -5,71 -9,99 08/26/15 CH07-04-1.2 Heidelberg

2 47,10 0,90 -5,57 -10,71 08/26/15 CH07-04-1.1 Heidelberg

2 48,00 0,90 -6,06 -10,46 08/26/15 CH07-04-1 Heidelberg

3 49,00 49,00 -6,66 -10,74 08/26/15 CH07-03-2 Heidelberg

3 49,90 0,90 -5,82 -10,59 08/26/15 CH07-03-1.19 Heidelberg

3 50,80 0,90 -5,11 -11,01 08/26/15 CH07-03-1.18 Heidelberg

3 51,70 0,90 -5,52 -9,10 08/26/15 CH07-03-1.17 Heidelberg

3 52,60 0,90 -5,77 -10,10 08/26/15 CH07-03-1.16 Heidelberg

3 53,70 1,10 -5,55 -9,59 08/26/15 CH07-03-1.15 Heidelberg

3 54,60 0,90 -5,75 -9,91 08/26/15 CH07-03-1.14 Heidelberg

3 55,50 0,90 -5,28 -10,62 08/26/15 CH07-03-1.13 Heidelberg

3 56,40 0,90 -5,28 -9,42 08/26/15 CH07-03-1.12 Heidelberg

3 57,30 0,90 -5,34 -10,36 08/26/15 CH07-03-1.11 Heidelberg

3 58,20 0,90 -6,47 -10,40 08/28/15 CH07-03-1.10 Heidelberg

3 59,10 0,90 -5,24 -10,21 08/28/15 CH07-03-1.9 Heidelberg

3 60,00 0,90 -7,00 -10,77 08/28/15 CH07-03-1.8 Heidelberg

3 60,90 0,90 -5,35 -10,22 08/28/15 CH07-03-1.7 Heidelberg

3 61,80 0,90 -4,63 -9,21 08/28/15 CH07-03-1.6 Heidelberg

3 62,70 0,90 -5,12 -10,02 08/28/15 CH07-03-1.5 Heidelberg

3 63,60 0,90 -5,56 -9,27 08/28/15 CH07-03-1.4 Heidelberg

3 64,50 0,90 -7,00 -9,42 08/28/15 CH07-03-1.3 Heidelberg

3 65,40 0,90 -4,95 -9,42 08/28/15 CH07-03-1.2 Heidelberg

3 66,30 0,90 -5,61 -8,99 08/28/15 CH07-03-1.1 Heidelberg

3 67,20 0,90 -6,44 -9,26 08/28/15 CH07-03-1 Heidelberg

4 70,00 70,00 -4,69 -10,63 08/28/15 CH07-02-39.8 Heidelberg

4 70,90 0,90 -4,14 -10,76 08/28/15 CH07-02-39.7 Heidelberg

4 71,80 0,90 -4,05 -10,27 08/28/15 CH07-02-39.6 Heidelberg

4 72,70 0,90 -4,75 -10,36 08/27/15 CH07-02-39.5 Heidelberg

4 73,60 0,90 -5,78 -10,32 08/27/15 CH07-02-39.4 Heidelberg

4 74,50 0,90 -6,10 -10,25 08/27/15 CH07-02-39.3 Heidelberg

4 75,40 0,90 -6,08 -9,57 08/27/15 CH07-02-39.2 Heidelberg

4 76,30 0,90 -7,13 -9,51 08/27/15 CH07-02-39.1 Heidelberg

4 77,20 0,90 -7,06 -9,23 08/27/15 CH07-02-39 Heidelberg

4 78,10 0,90 -6,89 -9,12 08/27/15 CH07-02-28.6 Heidelberg

4 79,00 0,90 -6,80 -11,59 08/27/15 CH07-02-28.5 Heidelberg

4 79,90 0,90 -7,47 -10,85 08/27/15 CH07-02-28.4 Heidelberg

4 80,80 0,90 -6,14 -10,84 08/27/15 CH07-02-28.3 Heidelberg

4 81,70 0,90 -6,25 -11,12 08/27/15 CH07-02-28.2 Heidelberg

4 82,60 0,90 -5,59 -10,93 08/27/15 CH07-02-28.1 Heidelberg

4 83,50 0,90 -6,35 -11,37 08/27/15 CH07-02-28 Heidelberg

4 84,40 0,90 -5,15 -11,50 08/27/15 CH07-02-27.10 Heidelberg

4 85,30 0,90 -4,77 -11,61 08/27/15 CH07-02-27.9 Heidelberg

4 86,20 0,90 -5,62 -11,26 08/27/15 CH07-02-27.8 Heidelberg

4 87,10 0,90 -6,48 -11,11 08/27/15 CH07-02-27.7 Heidelberg

4 88,00 0,90 -5,83 -10,66 08/27/15 CH07-02-27.6 Heidelberg

4 88,90 0,90 -6,16 -10,76 08/27/15 CH07-02-27.5 Heidelberg

4 89,80 0,90 -6,68 -10,95 08/27/15 CH07-02-27.4 Heidelberg

4 90,70 0,90 -6,90 -11,03 08/27/15 CH07-02-27.3 Heidelberg

4 91,60 0,90 -5,22 -10,86 08/27/15 CH07-02-27.2 Heidelberg

4 92,50 0,90 -5,72 -7,87 08/27/15 CH07-02-27.1 Heidelberg

4 93,40 0,90 -5,38 -7,14 08/27/15 CH07-02-27 Heidelberg

5 95,00 95,00 -3,70 -6,34 08/27/15 CH07-01-29 Heidelberg

5 95,31 0,31 -3,59 -6,66 08/27/15 CH07-01-28 Heidelberg

5 95,62 0,31 -4,41 -7,95 08/27/15 CH07-01-27 Heidelberg

5 95,92 0,31 -4,95 -8,98 08/27/15 CH07-01-26 Heidelberg

5 96,23 0,31 -5,12 -8,29 08/27/15 CH07-01-25 Heidelberg

5 96,54 0,31 -4,96 -7,99 08/13/15 CH7-01 24 Heidelberg

5 96,85 0,31 -4,66 -8,32 08/13/15 CH7-01 23 Heidelberg

5 97,15 0,31 -5,04 -9,14 08/13/15 CH7-01 22 Heidelberg

5 97,46 0,31 -5,16 -9,47 08/13/15 CH7-01 21 Heidelberg

5 97,77 0,31 -5,45 -9,81 08/13/15 CH7-01 19 Heidelberg

5 98,08 0,31 -5,40 -9,85 08/13/15 CH7-01 17 Heidelberg

5 98,38 0,31 -5,49 -9,80 08/13/15 CH7-01 16 Heidelberg

5 98,69 0,31 -5,25 -9,87 08/13/15 CH7-01 15 Heidelberg

5 99,00 0,31 -4,89 -9,25 08/13/15 CH7-01 13 Heidelberg

5 99,31 0,31 -4,72 -8,82 08/13/15 CH7-01 12 Heidelberg

5 99,62 0,31 -4,51 -8,49 08/13/15 CH7-01 11 Heidelberg

5 99,92 0,31 -4,63 -8,91 08/13/15 CH7-01 10 Heidelberg

5 100,23 0,31 -4,67 -9,22 08/13/15 CH7-01 09 Heidelberg

5 100,54 0,31 -4,76 -9,35 08/13/15 CH7-01 08 Heidelberg

5 100,85 0,31 -4,91 -9,49 08/13/15 CH7-01 07 Heidelberg

5 101,15 0,31 -4,88 -9,36 08/13/15 CH7-01 06 Heidelberg

5 101,46 0,31 -4,92 -9,34 08/13/15 CH7-01 05 Heidelberg

5 101,77 0,31 -4,91 -8,77 08/13/15 CH7-01 04 Heidelberg

5 102,08 0,31 -5,28 -8,38 08/13/15 CH7-01 03 Heidelberg

5 102,38 0,31 -5,03 -8,02 08/13/15 CH7-01 02 Heidelberg

5 103,00 0,62 -5,26 -7,33 08/13/15 CH7-01 01 Heidelberg
